# Comparison of the effect of iguratimod and hydroxychloroquine on regulatory B cells in the treatment of primary Sjögren’s syndrome

**DOI:** 10.3389/fmed.2025.1573973

**Published:** 2025-07-03

**Authors:** Jin-Mei Zou, Li Yang, Jia-Ang Luo, Yan Ren, Si-Yin Li, Yu Zhang, Jian-Ling Dong, Dai-Hua Deng, Yuan-Piao Ni, Min Li, Xiao-Shuang Yin, Jing Yang

**Affiliations:** ^1^Department of Rheumatology and Immunology, Mianyang Central Hospital, School of Medicine, University of Electronic Science and Technology of China, Mianyang, China; ^2^First Veterans Hospital of Sichuan Province, Chengdu, China

**Keywords:** primary Sjögren’s syndrome, regulatory B cells, iguratimod, hydroxychloroquine, treatment

## Abstract

**Objectives:**

This study aimed to evaluate the efficacy of iguratimod (IGU) and hydroxychloroquine (HCQ) in the treatment of primary Sjögren’s syndrome (pSS).

**Methods:**

This was a randomised controlled study. A total of 60 patients with pSS in Mianyang Central Hospital were recruited between December 2020 and December 2022. They were randomly divided into two groups: the IGU group and the HCQ group. Treatment in the IGU group was as follows: ≤10 mg of prednisone per day, 25 mg of IGU twice a day; treatment in the HCQ group was as follows: ≤10 mg of prednisone per day, 0.2 g of HCQ twice a day. The EULAR Sjögren’s Syndrome Disease Activity Index (ESSDAI) and the European League against Desiccation Sjögren’s Syndrome Patient Reported Index (ESSPRI) were used to assess disease activity.

**Results:**

After 6 months of treatment, the levels of immunoglobulin G (IgG) and ESSPRI in the IGU group were significantly lower than those in the HCQ group and the levels of CD19^+^CD5^+^CD1d^+^ B cells were significantly higher than those in the HCQ group (*p* < 0.05). Compared with baseline, the serum IgG level, erythrocyte sedimentation rate (ESR), B lymphocytes, ESSDAI, ESSPRI and Functional Assessment of Chronic Illness Therapy (FACIT) were significantly decreased and CD19^+^CD5^+^CD1d^+^ B cells were significantly increased in the IGU group after 6 months of treatment. In the HCQ group, C-reactive protein, ESR, ESSDAI, ESSPRI and FACIT were significantly decreased; there was no significant difference in regulatory B cells before and after treatment.

**Conclusion:**

Both IGU and HCQ can reduce the disease activity and fatigue score of patients with pSS. However, IGU was superior to HCQ in reducing IgG levels. Furthermore, IGU can affect the levels of peripheral blood B lymphocytes and CD19^+^CD5^+^CD1d^+^ B cells.

## Introduction

Primary Sjögren’s syndrome (pSS) is an autoimmune disease characterised by a large number of lymphocytes and plasma cells invading the lacrimal gland, salivary gland and other exocrine glands. In China, the prevalence of pSS is 0.33–0.77%, with a predominance in women, and the peak onset age is 40–50 years. The male-to-female ratio is 1:9–1:20 (1). Its pathogenesis is complex and involves the combined effects of genetic susceptibility, environmental factors and immune disorders (2, 3). In terms of genetic inheritance, studies have shown that certain specific human leukocyte antigen (HLA) genes are associated with the onset of pSS; genes in the non-HLA region are also involved. Moreover, the susceptibility genes vary among different populations (4). Among environmental factors, viral infections, especially Epstein–Barr virus infection, are considered major risk factors. The Epstein–Barr virus may trigger pSS through mechanisms such as B-cell activation, molecular mimicry and the protective role of tertiary lymphoid structures (TLS) in maintaining abnormal viral infections (5). Sex hormones also play a role in the pathogenesis. Oestrogens may increase the risk of developing the disease by enhancing inflammatory responses and elevating the levels of autoantibodies. Therefore, the incidence rate in women is much higher than in men (6). In terms of immune response, the activation of both innate and adaptive immunity is thought to play a major role in the pathogenesis of pSS. The dysfunction or abnormal activation of these immune cells can lead to lymphocyte infiltration and the production of autoantibodies, ultimately promoting the development of pSS. Specifically, the imbalance of the cytokine network is a key factor in the pathogenesis of pSS. For example, the overproduction of pro-inflammatory cytokines, such as interleukin (IL)-6, IL-17 and tumour necrosis factor alpha (TNF-*α*), as well as the insufficient secretion of anti-inflammatory cytokines, such as IL-10 and transforming growth factor beta (TGF-*β*), can exacerbate inflammatory responses and lead to tissue damage (7). In addition, the abnormal activation of signalling pathways, such as the interferon gamma (IFN-*γ*) signalling pathway, nuclear factor kappa-light-chain-enhancer of activated B cells (NF-κB) signalling pathway and Toll-like receptor (TLR) signalling pathway, also plays a key role in the pathogenesis of pSS. These signalling pathways are involved in the regulation of immune cell functions and the production of inflammatory mediators. For example, the IFN-*γ* signalling pathway can stimulate salivary gland epithelial cells to secrete various inflammatory factors and chemokines and activate the NF-κB and JAK/STAT signalling pathways, mediating the inflammatory process in the salivary glands of patients with pSS (8). The formation of TLS in the salivary glands of patients with pSS is an important pathological feature of the disease. These structures are formed and maintained by the continuous crosstalk between immune cells and non-immune cells. The lack of IL-17 signalling indirectly promotes the expansion and abnormal activity of TLS, leading to a strong IFN-*γ* response (9), which in turn causes abnormal activation of the interferon pathway and leads to immune system dysregulation, making disease progression more severe. The pathological changes in pSS are mainly characterised by lymphocyte infiltration in the minor salivary glands, with T and B lymphocytes being the predominant cell types involved, and only about 10% infiltration of macrophages and dendritic cells (10). Among these cells, B cells play a central role in the pathogenesis of pSS. The over-activation of B cells is one of the typical features of pSS, which is clinically manifested as hypergammaglobulinemia, the production of various autoantibodies, high levels of B-cell-activating factor, B-cell subset disorders and a significant increase in the risk of lymphoma, with approximately 5% of patients with pSS eventually progressing to lymphoma (11).

Human peripheral blood B cells are derived from haematopoietic stem cells in the bone marrow. Studies have shown that they can be divided into different subgroups based on five different surface molecules: CD19, CD45, CD24, CD38 and CD27. These subgroups include regulatory B cells (Bregs, CD19^+^CD24^hi^CD38^hi^), naive B cells (CD19^+^CD24^int^CD38^int^), two types of memory B cells (CD19^+^CD38^−^CD24^+^ and CD19^+^CD38^−^CD27^+^) and two types of plasmablasts (CD19^+^CD24^−^CD38^hi^ and CD19^+^CD27^hi^CD38^hi^) (12). Among them, Bregs are a group of B cells that negatively impact immune responses. They can exert anti-inflammatory effects by inhibiting T- and B-cell responses and producing cytokines, such as IL, TGF-*β* and granzyme B, and may play a role in suppressing excessive autoimmunity in pSS (13). Based on different surface markers, Bregs in the human body are mainly divided into different phenotypes, such as CD19^+^CD25^hi^CD71^hi^CD73-B cells, CD19^+^CD24^hi^CD38^hi^ B cells, CD19^+^CD24^+^CD27^+^B cells, CD19^+^CD5^+^CD1d^hi^ B cells and CD19^+^CD38^+^CD1d^+^IgM^+^CD147^+^ B cells (14). All play a role in negatively regulating immunity. Current research on Breg cells mainly focuses on three phenotypes: CD19^+^CD24^hi^CD38^hi^ B cells, CD19^+^CD24^+^CD27^+^ B cells and CD19^+^CD5^+^CD1d^hi^ B cells. One study (15) found that Bregs are more elevated in patients with pSS with clinical activity and extraglandular manifestations.

Primary Sjögren’s syndrome treatment includes local and systemic treatment. Local treatment mainly uses drugs that promote the secretion of glands or artificial saliva or tears to improve dry mouth and dry eye symptoms. Immunomodulatory drugs are often used according to the situation of system damage, and the recommended drug is hydroxychloroquine (HCQ) (16, 17). Previous literature has reported that HCQ can reduce dry mouth symptoms, improve fatigue and reduce C-reactive protein (CRP) and erythrocyte sedimentation rate (ESR) levels in patients with pSS (18–21). However, pSS is a highly heterogeneous disease; different immunosuppressant treatments are needed according to the different organ involvement of patients. The molecular structure of iguratimod (IGU) is n-[7-[(methanesulfonyl) amino]-4-oxo-6-phenoxy-4 h-1-benzopyran-3-yl]-formamide. It can selectively inhibit cyclooxygenase-2 (Cox-2) and inhibit the production of cytokines, such as IFN-*γ*, IL-1, IL-6, TNF-*α*, IL-17, immunoglobulin (Ig) M and IgG, substantially reducing the inflammatory response (22, 23). It was approved in China and Japan in 2012 for the treatment of rheumatoid arthritis (RA). Iguratimod has been subsequently used for the treatment of other autoimmune diseases, such as pSS, IgG4-related diseases and lupus nephritis (24, 25). Multiple randomised controlled trials and meta-analyses have shown that IGU achieves significant effects in pSS; in particular, IGU was better than HCQ at reducing the level of Ig (18, 26, 27). Therefore, the 2020 diagnosis and treatment norms of pSS in China recommended IGU as an immunosuppressant (28).

Several studies have shown that IGU has achieved certain efficacy in the treatment of pSS, which can effectively inhibit the activation of B cells, reduce Ig levels, avoid multiple organ damage caused by hypergammaglobulinemia and reduce the EULAR Sjögren’s Syndrome Disease Activity Index (ESSDAI) and the European League Against Desiccation Sjögren’s Syndrome Patient Reported Index (ESSPRI) (22, 27, 29, 30). However, there is no study examining whether IGU affects Bregs in patients with pSS. The purpose of this study is to evaluate the effect of IGU and HCQ in the treatment of pSS and to analyse the influence of these two drugs on Bregs in peripheral blood.

## Materials and methods

### Patients

**Figure 1:**
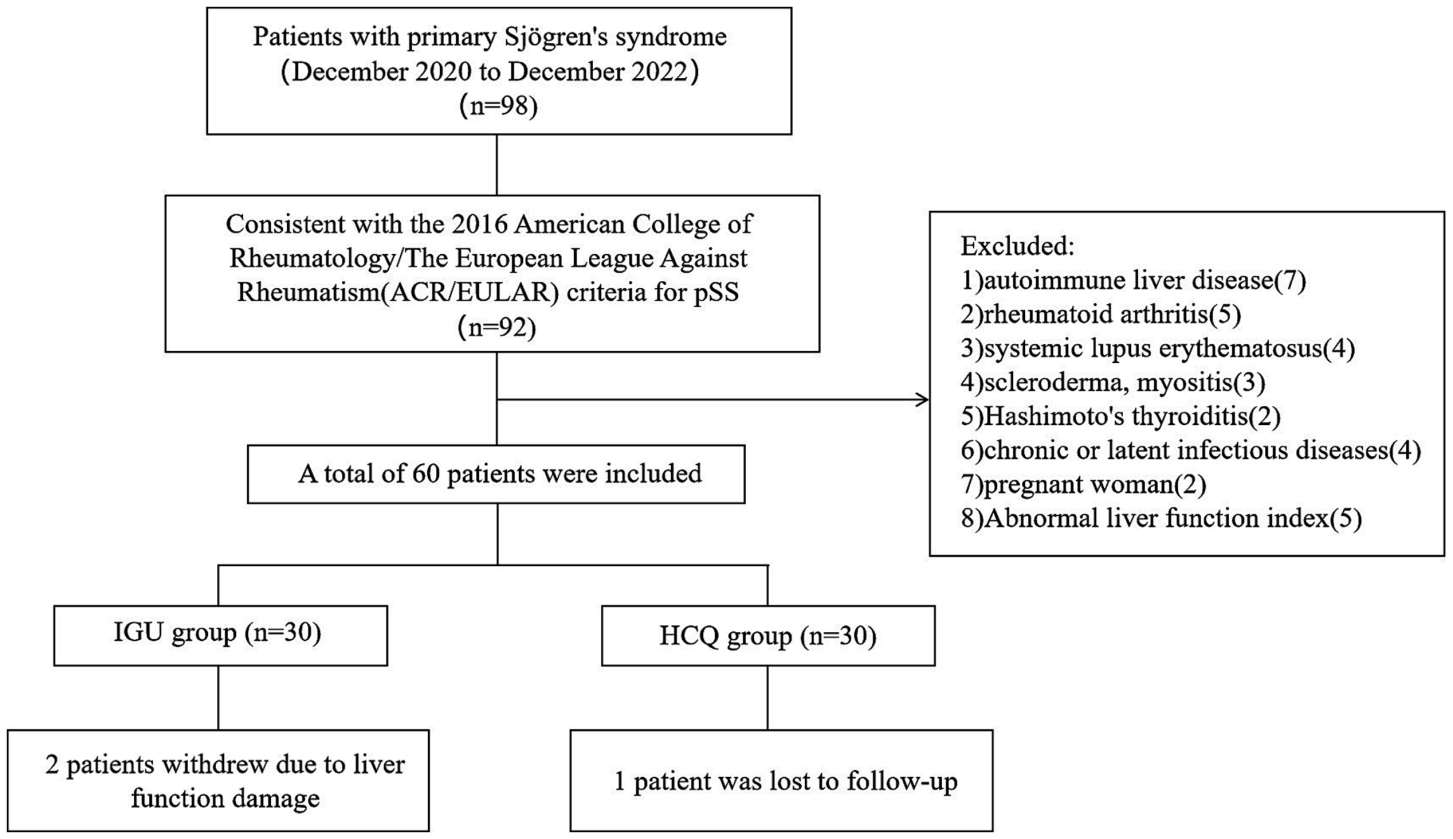
Study population screening flow chart.

This was a prospective, single-centre, randomised controlled study. Patients from the Mianyang Central Hospital were recruited between December 2020 and December 2022. The protocol was approved by the Ethics Committee of the Mianyang Central Hospital (S-2020-048), and written informed consent was obtained from all patients before they participated in the study. This study has been registered at the registry https://www.isrctn.com with the number: ISRCTN15824224. According to the G*Power software tool (Heinrich-Heine-Universität Düsseldorf, Düsseldorf, Germany), the efficacy of statistical analysis is required to be within the acceptable range of 70–80%, and the samples of the two groups should be >23 cases. A total of 60 patients who fulfilled the 2016 American College of Rheumatology/The European League Against Rheumatism criteria for pSS were recruited for this study (Figure 1). The eligibility criteria were as follows: aged 18–65 years; no glucocorticoids, immunosuppressants or biological agents within 3 months before baseline; consent to contraception during the trial and within 3 months after the end of the trial. The exclusion criteria were as follows: patients with other immune system diseases, such as autoimmune liver disease, RA, systemic lupus erythematosus, scleroderma, myositis or Hashimoto’s thyroiditis; patients with serious organ involvement, such as severe pericardial effusion (echocardiography showing pericardial effusion thickness >10 mm), pulmonary interstitial lesions (high-resolution computed tomography showing ground-glass opacity or honeycomb lung), renal tubular acidosis (serum bicarbonate level >30 mmol/L and a urine pH value persistently >6.0) or atrophic gastritis (endoscopy showing gastric mucosal atrophy); patients with underlying cardiac, pulmonary, renal, gastrointestinal or metabolic conditions; patients with chronic or latent infectious diseases or a history of malignancy, mental diseases or alcohol abuse; pregnant and lactating women; patients with the following abnormal indicators – haemoglobin ≤90 g/L, platelet count <100 × 10^9^/L, white blood cell count <3.0 × 10^9^/L or >14 × 10^9^/L, estimated glomerular filtration rate ≤45 mL/min/1.73 m^2^, total bilirubin >1.5 × upper limit of normal (ULN), aspartate aminotransferase and alanine aminotransferase both >1.5 × ULN.

### Reagents and materials

The flow cytometry tubes were purchased from Zhejiang Gongdong Medical Instruments Co., Ltd., China. The PK-31 diluent for blood cell analysis was obtained from Sysmex. Foetal bovine serum (1%) was sourced from GIBCO life, and paraformaldehyde (1%) was acquired from Chengdu Kelong Chemicals Co., Ltd., China. The pipette tips were purchased from Axygen, Mexico (0.5–10 μL), Haimen Guolong Laboratory Instruments, China (10–200 μL), and Jiangsu Kangjian Medical, China (100–1,000 μL). The antibodies used for flow cytometry include CD5 FITC (Catalogue Number: 555352, BD Biosciences, USA), CD24 PE (Catalogue Number: 983602, BioLegend, USA), CD19 ECD (Catalogue Number: 6604551, Beckman Coulter, USA), CD1d PC7 (Catalogue Number: 350308, BioLegend), CD38 APC-A750 (Catalogue Number: A86049, Beckman Coulter), CD27 PacBlue (Catalogue Number: B30635, Beckman Coulter) and CD45 BV510 (Catalogue Number: 563204, BD Biosciences). The red blood cell lysing solution OptiLyse C was obtained from Beckman Coulter (Catalogue Number: A11895).

### Study design

The patients were randomly assigned to an IGU group (*n* = 30) or an HCQ group (*n* = 30) at a ratio of 1:1. All the patients were allowed to receive <0 mg of prednisone per day and vitamin D and calcium for 24 weeks to prevent osteoporosis; 25 mg of IGU was administered orally twice a day in the IGU group, and 0.2 g of HCQ was administered orally twice a day in the HCQ group. However, other immunosuppressants, such as methotrexate, leflunomide, mycophenolate mofetil and cyclosporine, and biological agents, such as belimumab and rituximab, were not allowed during the treatment period.

Disease activity was evaluated by ESSDAI and ESSPRI. A 10-cm visual analogue scale was used for rating the patient global assessment. The Functional Assessment of Chronic Illness Therapy (FACIT) was used to assess the fatigue degree. Clinical and laboratory data were collected at the same time. All variables were assessed at baseline and week 24.

### Flow cytometric analysis

**Figure 2:**
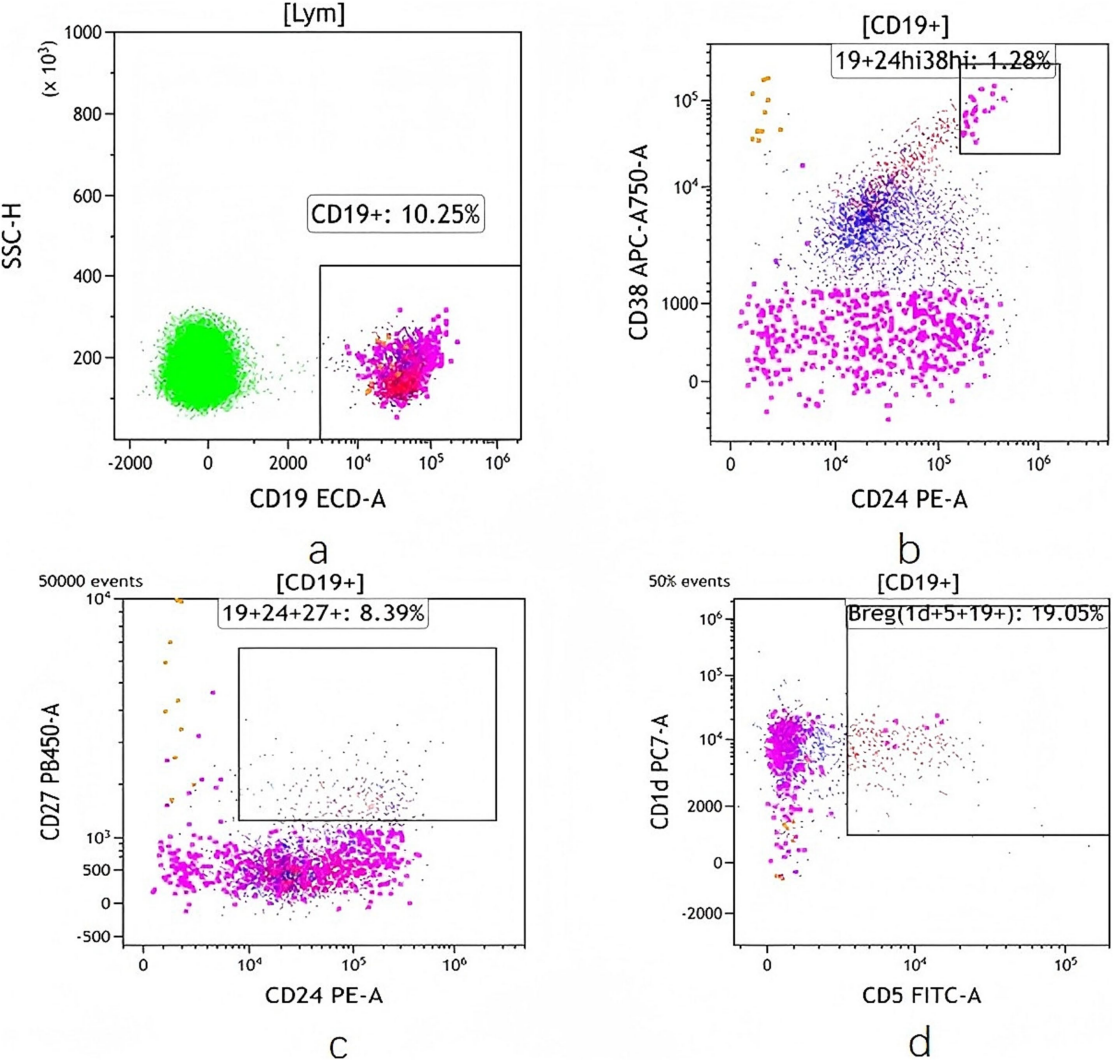
Frequency of Breg cells detected by flow cytometry. **(a)** CD19 + Bcell. **(b)** CD19 + CD24hi CD38hi Bcell. **(c)** CD19 + CD24 + CD27 + Bcell. **(d)** CD19 + CD5 + CD1d + Bcell.

The B lymphocytes and Bregs (CD19^+^CD24^hi^CD38^hi^, CD19^+^CD24^+^CD27^+^and CD19^+^ CD5^+^CD1d^+^ B cells) were detected by flow cytometry, and the values were expressed by the percentage of Breg cells in B lymphocytes. Peripheral blood samples (5-ml EDTA anticoagulant tube) from all the patients were collected at baseline and week 24. Peripheral blood mononuclear cells were prepared and stained with the following antibodies: CD5 FITC, PE anti-human CD24, CD19-ECD, PE/Cyanine7 anti-human CD1d, CD38-APC-ALexarFluor 750, CD27-Pacific Blue, BV510 Mouse Anti-Human CD45 (CD5FITC 20ul/test, CD24PE 5 μL/test, CD19ECD 10 μL/test, CD1dPC7 5 μL/test, CD38APC-A750 10 μL/test, CD27PacBlue 5 μL/test, CD45BV510 5 μL/test) (Beckman Coulter). The samples were analysed on a DXflex 13 colour flow cytometer (Beckman Coulter). The gating strategy for Breg cells can be found in the Figure 2.

### Statistical methods

All data were analysed using SPSS 26.0 software (IBM Corporation, Armonk, NY, USA). When the measurement data obeyed the normal distribution, they were described by the mean ± standard deviation and the paired *t*-test was used. If the data did not obey the normal distribution, the median (interquartile interval) was used to describe them and the nonparametric test was used to compare the differences. The *χ^2^* test was used for counting data, and a *p*-value of <0.05 was considered statistically significant.

## Results

All the patients were female. Two patients in the IGU group withdrew due to liver function damage, one patient in the HCQ group was lost to follow-up and 28 and 29 patients in these two groups completed the 24-week treatment, respectively (Figure 1). Before treatment, there was no statistical difference between the IGU group and the HCQ group in the baseline data. The clinical and demographic characteristics of the study participants are shown in Table 1.

**Table 1:** Demographic and other baseline characteristics of the study population.

Characteristic	IGU group	HCQ group	*t/Z*	Sig	*p* value
age $\left(\bar{x}\pm s\right)$	51.17 ± 11.35	55.37 ± 12.99	−1.334	0.188	*p* > 0.05
IgG (g/L)	17.89 ± 4.83	17.33 ± 6.84	0.367	0.715	*p* > 0.05
WBC 109/L	4.47 (1.93)	4.85 (2.52)	−0.372	0.824	*p* > 0.05
Hb (g/L)	118.27 ± 14.07	114.50 ± 20.73	0.823	0.414	*p* > 0.05
PLT 109/L	159.47 ± 61.71	187.47 ± 80.51	−1.512	0.136	*p* > 0.05
CRP	4.37 (7.45)	3.08 (6.48)	0.837	0.215	*p* > 0.05
ESR	28.50 (24.00)	31.00 (49.00)	−0.872	0.317	*p* > 0.05
ALT	22.50 (16.00)	18.50 (38.00)	0.974	0.281	*p* > 0.05
AST	24.50 (10.00)	27.00 (18.00)	−1.032	0.152	*p* > 0.05
GFR	77.82 ± 11.87	73.21 ± 16.82	1.226	0.225	*p* > 0.05
Lymphocyte	20.43 ± 10.81	29.65 ± 7.61	−3.819	0.002	*p* < 0.05
B cell	15.22 (9.99)	13.18 (6.81)	1.011	0.154	*p* > 0.05
CD19 + CD5 + CD1d+	22.92 ± 12.46	27.88 ± 8.49	−1.803	0.077	*p* > 0.05
CD19 + CD24 + CD27+	7.47 (5.81)	7.94 (6.27)	−0.521	0.455	*p* > 0.05
CD19 + CD24hi CD38hi	7.56 (14.63)	4.79 (4.19)	2.247	0.021	*p* < 0.05
EESDAI	4.00 (3.00)	4.00 (6.00)	−1.071	0.094	*p* > 0.05
ESSPRI	4.36 ± 1.65	4.50 ± 1.07	−0.401	0.69	*p* > 0.05
FACIT	23.40 ± 8.97	22.33 ± 6.15	0.537	0.593	*p* > 0.05

IgG, immune globulin G; Hb, hemoglobin; CRP, C reactive protein; ESR, Erythrocyte sedimentation rate; ALT, Alanine aminotransferase; AST, aspartate transaminase; GFR, Glomerular filtration rate; ESSDAI, disease activity index; ESSPRI, the European League against desiccation pSS patient reported index; FANCIT, the functional assessment of chronic illness therapy, *p* < 0.05 represents significant difference between IGU group versus HCQ group.

After 6 months of treatment, the IgG levels and ESSPRI scores in the IGU group were significantly lower than those in the HCQ group; the number of CD19^+^CD5^+^CD1d^+^ B cells was higher in the IGU group than in the HCQ group (Table 2).

**Table 2:** Comparison with IGU and HCQ group after 6 months of treatment.

Characteristic	IGU group	HCQ group	*t/Z*	Sig	*p* value
IgG (g/L)	13.84 ± 1.70	16.63 ± 5.10	−2.756	0.008	*p* < 0.05
WBC 109/L	4.65 (1.90)	5.55 (1.75)	−1.504	0.135	*p* > 0.05
Hb (g/L)	119.00 ± 11.28	119.10 ± 12.88	−0.031	0.979	*p* > 0.05
PLT 109/L	158.21 ± 40.85	181.69 ± 55.81	−1.317	0.156	*p* > 0.05
CRP	3.35 (2.58)	3.50 (1.18)	1.067	0.174	*p* > 0.05
ESR	13.50 (10.00)	18.50 (10.00)	−1.032	0.256	*p* > 0.05
ALT	23.50 (12.00)	27.00 (13.00)	−1.024	0.327	*p* > 0.05
AST	25.00 (12.00)	34.00 (9.00)	−1.712	0.078	*p* > 0.05
GFR	76.81 ± 10.38	73.94 ± 12.31	0.967	0.715	*p* > 0.05
Lymphocyte	26.73 ± 43.35	30.33 ± 5.13	−1.461	0.075	*p* > 0.05
B cell	14.54 (7.42)	14.84 (5.24)	−1.062	0.092	*p* > 0.05
CD19 + CD5 + CD1d+	31.13 ± 9.22	25.33 ± 7.13	3.717	0.012	*p* < 0.05
CD19 + CD24 + CD27+	8.15 (10.38)	7.43 (7.55)	1.028	0.164	*p* > 0.05
CD19 + CD24hi CD38hi	8.15 (13.87)	6.63 (3.22)	1.441	0.070	*p* > 0.05
EESDAI	1.00 (2.00)	2.00 (1.00)	−0.973	0.314	*p* > 0.05
ESSPRI	1.48 ± 0.72	1.86 ± 0.64	−2.08	0.042	*p* < 0.05
FACIT	13.00 ± 5.06	13.46 ± 4.03	−0.38	0.705	*p* > 0.05

*p* < 0.05 represents significant difference between IGU group versus HCQ group.

**Figure 3:**
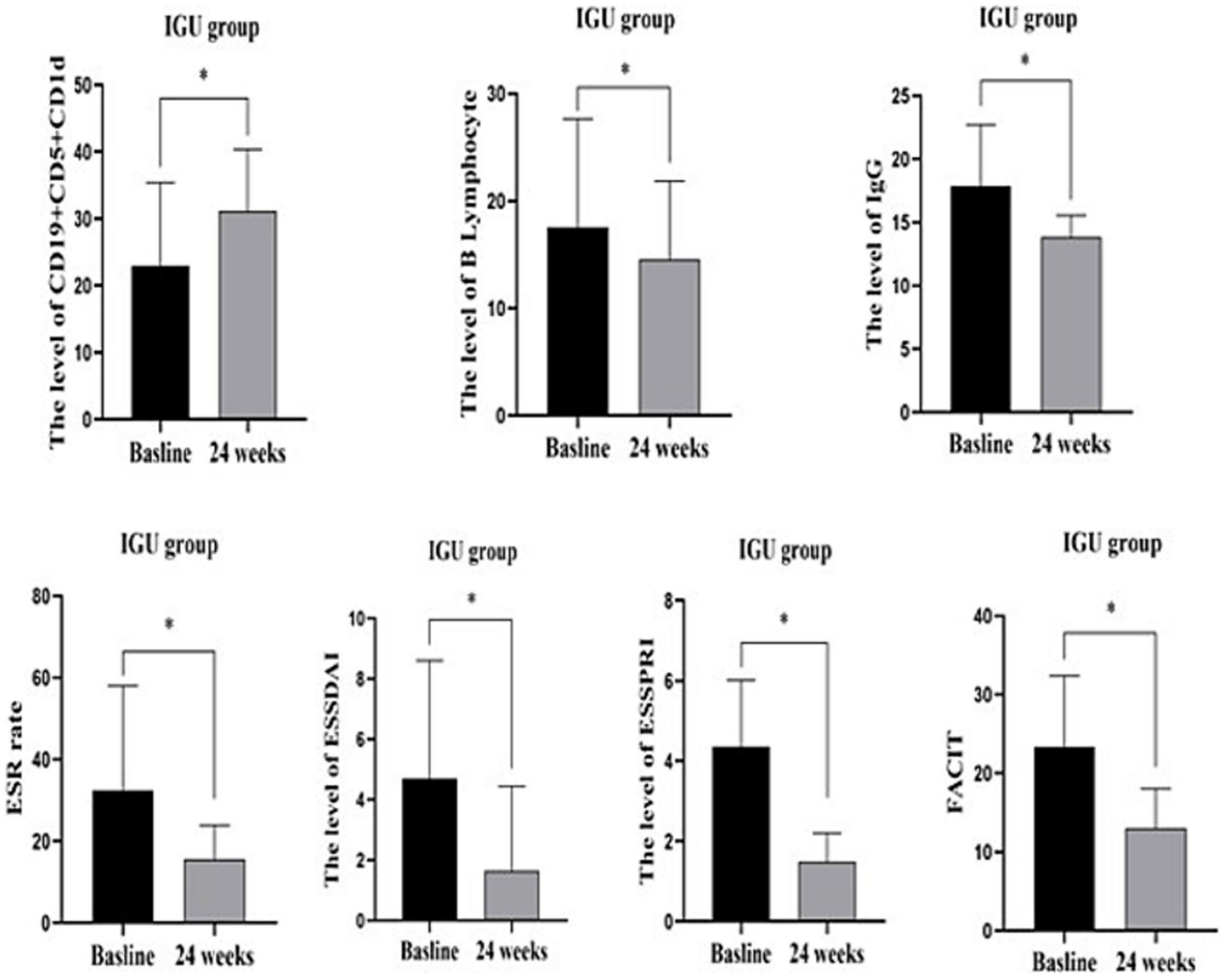
IGU group before and after 24 weeks treatment: CD19^+^CD5^+^CD1d^+^ was significantly increased, while B lymphocytes, serum IgG, ESR, ESSDAIL, ESSPRI, FACIT were significantly decreased compared with the baseline. *: *p* < 0.05 represents significant difference post-treatment versus baseline in IGU group. IgG, immune globulin G; CRP, C reactive protein; ESR, Erythrocyte sedimentation rate; ESSDAIL, disease activity index; ESSPRI, the European League against desiccation pSS patient reported index; FANCIT, the functional assessment of chronic illness therapy.

In the IGU group, compared with baseline, serum IgG, ESR, B lymphocytes, ESSDAI, ESSPRI and FACIT were significantly decreased, whereas the number of CD19^+^CD5^+^CD1d^+^ B cells was significantly increased after 6 months of treatment (Table 3; Figure 3).

**Table 3:** Comparison within IGU group before and after 6 months treatment.

Characteristic	Before treatment	After treatment	*t/Z*	*p* value
IgG (g/L)	17.89 ± 4.83	13.84 ± 1.70	5.502	*p* < 0.05
WBC 109/L	4.47 (1.93)	4.65 (1.90)	−0.524	*p* > 0.05
Hb (g/L)	118.27 ± 14.07	119.00 ± 11.28	−0.890	*p* > 0.05
PLT 109/L	159.47 ± 61.71	158.21 ± 40.85	0.686	*p* > 0.05
CRP	7.20 ± 7.76	6.72 ± 12.04	0.338	*p* > 0.05
ESR	4.37 (7.45)	3.35 (2.58)	3.247	*p* < 0.05
ALT	28.50 (24.00)	13.50 (10.00)	1.024	*p* > 0.05
AST	22.50 (16.00)	23.50 (12.00)	−0.481	*p* > 0.05
GFR	24.50 (10.00)	25.00 (12.00)	−0.813	*p* > 0.05
Lymphocyte	20.43 ± 10.81	26.73 ± 43.35	−0.817	*p* > 0.05
B cell	15.22 (9.99)	14.54 (7.42)	2.012	*p* < 0.05
CD19^+^CD5^+^CD1d^+^	22.92 ± 12.46	31.13 ± 9.22	−3.860	*p* < 0.05
CD19^+^CD24^+^CD27^+^	7.47 (5.81)	8.15 (10.38)	−1.725	*p* > 0.05
CD19^+^CD24hi CD38hi	7.56 (14.63)	8.15 (13.87)	−0.827	*p* > 0.05
EESDAI	4.00 (3.00)	1.00 (2.00)	4.723	*p* < 0.05
ESSPRI	4.36 ± 1.65	1.48 ± 0.72	11.604	*p* < 0.05
FACIT	23.40 ± 8.97	13.00 ± 5.06	10.324	*p* < 0.05

*p* < 0.05 represents significant difference post-treatment versus baseline in IGU group.

**Figure 4:**
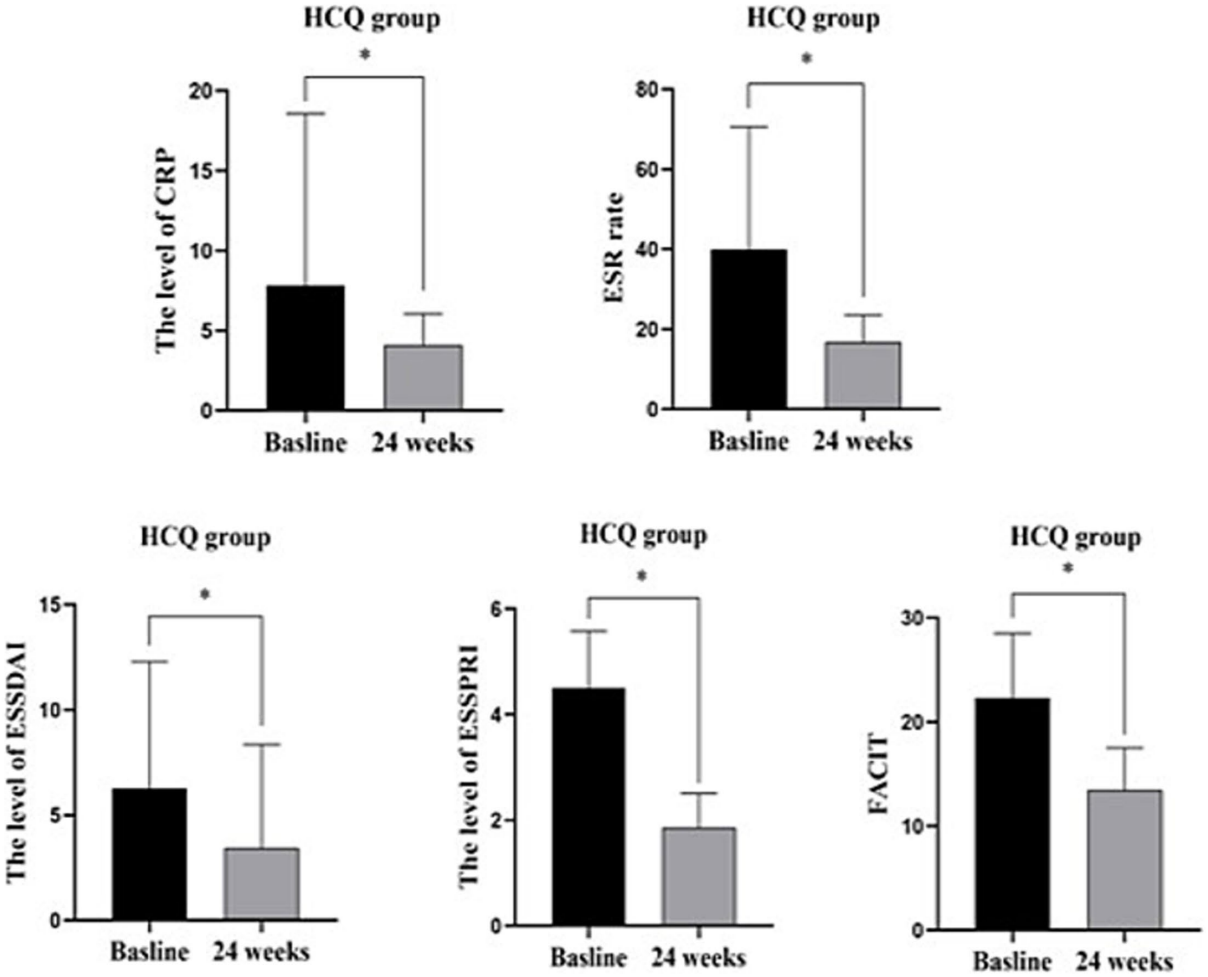
HCQ group before and 24 weeks treatment: CRP, ESR, ESSDAIL, ESSPRI, FACIT were significantly decreased compared with the baseline. There was no significant difference in other characteristics including B lymphocytes and Bregs. *: *p* < 0.05 represents significant difference post-treatment versus baseline in IGU group.

In the HCQ group, compared with baseline, CRP, ESR, ESSDAI, ESSPRI and FACIT were significantly decreased after 6 months of treatment. There was no significant difference in the rest of the characteristics, including B lymphocytes and Bregs (Table 4; Figure 4).

**Table 4:** Comparison within HCQ group before and after 6 months treatment.

Charactoristic	Before treatment	After treatment	*t/Z*	*p* value
IgG (g/L)	17.33 ± 6.84	16.63 ± 5.10	1.127	*p* > 0.05
WBC 109/L	4.85 (2.52)	5.55 (1.75)	−0.724	*p* > 0.05
Hb (g/L)	114.50 ± 20.73	119.10 ± 12.88	−1.134	*p* > 0.05
PLT 109/L	187.47 ± 80.51	181.69 ± 55.81	1.128	*p* > 0.05
CRP	3.08 (6.48)	3.50 (1.18)	1.143	*p* < 0.05
ESR	31.00 (49.00)	18.50 (10.00)	4.516	*p* < 0.05
ALT	18.50 (38.00)	27.00 (13.00)	−1.127	*p* > 0.05
AST	27.00 (18.00)	34.00 (9.00)	−1.005	*p* > 0.05
GFR	73.21 ± 16.82	73.94 ± 12.31	−0.343	*p* > 0.05
Lymphocyte	29.65 ± 7.61	30.33 ± 5.13	−0.191	*p* > 0.05
B cell	13.18 (6.81)	14.84 (5.24)	−0.962	*p* > 0.05
CD19^+^CD5^+^CD1d^+^	27.88 ± 8.49	25.33 ± 7.13	0.847	*p* > 0.05
CD19^+^CD24^+^CD27^+^	7.94 (6.27)	7.43 (7.55)	−0.424	*p* > 0.05
CD19^+^CD24hi CD38hi	4.79 (4.19)	6.63 (3.22)	0.881	*p* > 0.05
EESDAI	4.00 (6.00)	2.00 (1.00)	2.135	*p* < 0.05
ESSPRI	4.50 ± 1.07	1.86 ± 0.64	11.778	*p* < 0.05
FACIT	22.33 ± 6.15	13.46 ± 4.03	13.006	*p* < 0.05

*p* < 0.05 represents significant difference post-treatment versus baseline in HCQ group.

The adverse reactions in these two groups were mainly liver function abnormalities, gastrointestinal symptoms, rash, leukopenia, skin itching and headache; there was no significant difference between these two groups. Skin pigmentation occurred in two cases in the HCQ group, although no special treatment was provided (Table 5).

**Table 5:** Comparison of adverse reactions between IGU group and HCQ group.

Adverse reaction	IGU group (*n* = 28)	HCQ group (*n* = 29)	*χ*2	*p* value
Liver function abnormalities	3	1	0.259	0.61
Gastrointestinal symptoms	5	3	0.666	0.414
Rash	1	2	0.316	0.574
Leukopenia	1	1	0.001	0.98
Skin itching	2	3	0.183	0.669
Headache	2	1	0.390	0.532
Skin pigmentation	0	2	2.001	0.157

## Discussion

This study compared the therapeutic effects of IGU and HCQ in pSS and analysed their impacts on Bregs. The results demonstrated that after 24 weeks of treatment, IGU outperformed HCQ in reducing IgG levels and ESSPRI scores and significantly increased the levels of peripheral blood B lymphocytes and CD19^+^CD5^+^CD1d^+^ B cells. In contrast, the influence of HCQ on Bregs was not significant. After treatment, serum IgG levels, ESR, B lymphocytes, ESSDAI, ESSPRI and FACIT were significantly decreased compared with those before treatment in the IGU group. The CRP, ESR, ESSDAI, ESSPRI and FACIT values were significantly decreased, although the serum IgG level had no significant difference compared with those before treatment in the HCQ group.

The differences in therapeutic effects between IGU and HCQ may be attributed to their distinct mechanisms of action. Iguratimod, as a novel immunomodulatory drug, can selectively inhibit Cox-2, thereby reducing the production of prostaglandins in inflamed tissues and inhibiting the release of bradykinin from inflamed tissues, which further alleviates inflammatory responses. Moreover, IGU can selectively act on B cells, inhibiting their excessive proliferation and activation and modulating B cell functions; this leads to a reduction in autoantibody production and a decrease in Ig levels, thus mitigating autoimmune damage to the exocrine glands (31). In contrast, HCQ, a common immunomodulatory drug, exerts anti-inflammatory, immunomodulatory and immunosuppressive effects through multiple pathways, including the inhibition of TLR signalling, the blockade of antigen presentation, the inhibition of autophagy and the suppression of calcium mobilisation. Its impact on Ig production is relatively limited (32). This may explain why IGU is more effective than HCQ in reducing IgG levels.

In addition, this study focused on the levels of B lymphocytes and Bregs in the two groups. The study results showed that before treatment, the proportion of the CD19^+^CD24^hi^CD38^hi^ B cell subpopulation in the IGU group was significantly lower than that in the HCQ group, whereas the proportion of the CD19^+^CD5^+^CD1d^+^ B cell subpopulation reached the highest level in the IGU group. This unique phenomenon suggests that in patients with pSS, there may be considerable distribution differences among different Breg subpopulations, which may be closely related to the disease state or individual characteristics of the patients. Current studies have shown that there may be a certain degree of functional overlap and synergistic effects among different Breg subpopulations; however, the specific degree of overlap and interrelationships still need to be further explored in depth (33). After treatment, the level of B lymphocytes in the peripheral blood of the IGU group decreased, indicating that the level of B lymphocytes may be affected by IGU treatment. Moreover, the proportion of CD19^+^CD5^+^CD1d^+^ B cells in the IGU group increased significantly, suggesting that this subpopulation may be the key cell type for IGU to exert its immune regulatory effects. Compared with the HCQ group, the proportion of CD19^+^CD5^+^CD1d^+^ B cells in the two groups after treatment showed a significant difference, with the level in the IGU group being significantly higher than that in the HCQ group. In contrast, there was no significant difference in the proportions of the CD19^+^CD24^hi^CD38^hi^ and CD19^+^CD24^+^CD27^+^ B cell subpopulations. This may be because HCQ mainly exerts its effects by inhibiting TLR signalling and reducing the production of pro-inflammatory cytokines, without directly affecting the levels of Bregs. In comparison, IGU can interact with PDK1 to inhibit the Akt–mTOR-STAT3 signalling pathway, leading to the downregulation of the 5CZ6 gene expression and the upregulation of another gene expression downstream. This ultimately results in reduced T follicular helper cell differentiation and the decreased expression of helper function molecules, thereby enhancing the inhibitory effects on B cells. This mechanism may enhance the regulatory functions of Bregs to achieve a more balanced immune response (34). Previous studies have shown that HCQ can effectively relieve dry mouth symptoms in patients with pSS, improve fatigue status and reduce the levels of CRP and ESR; however, its impact on Bregs levels has not been fully investigated (18). Furthermore, multiple studies have demonstrated that IGU has notable therapeutic effects on improving IgG levels, ESSPRI scores and ESR in patients with pSS (26, 27, 31). This study further supports these findings and provides new insights into the different impacts of IGU and HCQ on Bregs, offering important references for future precision treatment strategies targeting pSS.

In summary, this study presents a detailed analysis of the differences in the impact of IGU and HCQ on Bregs in patients with pSS. Previous research has primarily focused on the clinical efficacy and general immune regulatory effects of these drugs; however, the effects on specific B cell subsets, especially Bregs, have not been fully investigated. Our study results highlight the potential role of Bregs in the therapeutic effects of IGU and suggest that targeting Bregs may be a promising strategy for treating pSS. This study also emphasises the importance of further exploring the mechanisms by which IGU and HCQ regulate B cells, which may help to develop more effective treatments for pSS.

## Limitations of this study

This study has several limitations. The sample size is relatively small, and further research is needed to expand the sample size, and the role of CD19^+^CD5^+^CD1d^+^ B cells in the occurrence and development of Sjogren’s syndrome needs further study. Furthermore, due to the limited funding of this study and the high cost of Bregs marker detection, healthy individuals were not included as controls in the study design. This limitation prevents us from comparing the proportion of Bregs in patients with pSS with that in healthy individuals. Future studies should include a healthy control group to provide a baseline reference for the proportion of Breg cells, thereby better elucidating the changes in Bregs before and after treatment.

## Conclusion

Both IGU and HCQ have beneficial effects in the treatment of pSS and can reduce the disease activity and fatigue score. However, IGU can reduce the level of IgG better than HCQ, and IGU also affects the levels of peripheral blood B lymphocytes and Bregs in patients with pSS.

## Data Availability

The original contributions presented in the study are included in the article/supplementary material, further inquiries can be directed to the corresponding authors.
